# Using optically pumped magnetometers to replicate task-related responses in next generation magnetoencephalography

**DOI:** 10.1038/s41598-024-56878-6

**Published:** 2024-03-18

**Authors:** Kristina Safar, Marlee M. Vandewouw, Julie Sato, Jasen Devasagayam, Ryan M. Hill, Molly Rea, Matthew J. Brookes, Margot J. Taylor

**Affiliations:** 1 Department of Diagnostic Imaging, Hospital for Sick Children, 555 University Avenue, Toronto, ON M5G 1X8 Canada; 2 Program in Neurosciences and Mental Health, Hospital for Sick Children, Toronto, Canada; 3 Autism Research Centre, Bloorview Research Institute, Holland Bloorview Kids Rehabilitation Hospital, Toronto, Canada; 4 Institute of Biomedical Engineering, University of Toronto, Toronto, Canada; 5 Sir Peter Mansfield Imaging Centre, School of Physics and Astronomy, University of Nottingham, Nottingham, UK; 6 Cerca Magnetics Limited, Castlebridge Office Village, Kirtley Drive, Nottingham, UK; 7 Department of Medical Imaging, University of Toronto, Toronto, Canada; 8 Department of Psychology, University of Toronto, Toronto, Canada

**Keywords:** Neuroscience, Magnetoencephalography

## Abstract

Optically pumped magnetometers (OPMs) offer a new wearable means to measure magnetoencephalography (MEG) signals, with many advantages compared to conventional systems. However, OPMs are an emerging technology, thus characterizing and replicating MEG recordings is essential. Using OPM-MEG and SQUID-MEG, this study investigated evoked responses, oscillatory power, and functional connectivity during emotion processing in 20 adults, to establish replicability across the two technologies. Five participants with dental fixtures were included to assess the validity of OPM-MEG recordings in those with irremovable metal. Replicable task-related evoked responses were observed in both modalities. Similar patterns of oscillatory power to faces were observed in both systems. Increased connectivity was found in SQUID-versus OPM-MEG in an occipital and parietal anchored network. Notably, high quality OPM-MEG data were retained in participants with metallic fixtures, from whom no useable data were collected using conventional MEG.

Magnetoencephalography (MEG) is a powerful means of measuring brain activity and plays an important role in both cognitive neuroscience research and clinical applications^1–4^. However, there are several challenges surrounding recording neural responses with MEG. Optically pumped magnetometers (OPMs) are the future of MEG—they offer a new wearable and commercially available means to measure the MEG signal, with substantial advances in signal strength, data quality, and tolerance to movement compared to conventional cryogenic MEG^5–7^. However, only a handful of studies have leveraged this innovative technology to characterize MEG data recordings^8–12^. Here we investigated the replicability of well-established MEG evoked responses, oscillatory power, and functional connectivity using OPMs and traditional MEG during an emotional face task.

To gain sufficient sensitivity to detect the very small magnetic fields from the brain, conventional MEG systems use an array of superconducting sensors, called superconducting quantum interference devices (SQUIDs), which are cryogenically cooled and housed in a “one-size-fits-all” thermally insulated liquid-helium dewar^3,6,13^. Due to thermal insulation, the sensors are sited at a fixed distance of ~ 2 cm from the surface of an adult scalp; this limited proximity reduces the available signal strength. In individuals with smaller head sizes, this sensor-to-brain distance markedly increases, resulting in reduced signal strength and spatial resolution^14^. The inflexible position of the sensors also results in inhomogeneous brain coverage, with increased distance between sensors typically observed for frontal and temporal lobes^15,16^. SQUID-MEG also restricts participant movement throughout the recording, as head motion relative to the sensors significantly degrades signal quality^17,18^. These limitations pose major challenges for data acquisition. In addition, participants with irremovable metallic devices, such as dental wires or braces, are often incompatible with the SQUID-MEG due to significant artefacts^19^.

Recent innovations in the type of sensors that can be employed for MEG are transforming this field. OPM-MEG measures neuromagnetic fields by manipulating the quantum properties of alkali atoms^20,21^. These sensors have comparable sensitivity to SQUID sensors^22,23^, however because cryogenic cooling is not required, the sensors can be situated closer to the head and adjusted according to participant head size. This offers significant improvements in signal sensitivity, data quality and retention, and the flexibility of sensor placement compared to traditional cryogenic MEG^6^. Another major advantage of OPM-MEG is that they are tolerant of head movement when background fields are controlled^24,25^; thus, data may still be recorded despite participant movement or even while participants engage in dynamic and interactive paradigms^26^. The movement of metallic fixtures, relative to the fixed sensors in cryogenic MEG, means that signals are irrecoverably contaminated. However, OPM sensors move with the implants, meaning that—in theory—it should be possible to capture data^6^. This has not yet been tested experimentally.

As a new technology, establishing the replicability and reliability of neural responses in OPM-MEG is essential. The viability of sensor level and source localized evoked and induced MEG responses has been demonstrated during sensorimotor, motor, and visuo-motor tasks in both a single participant and a small number of participants^7,9,10,16^. However, few studies have directly compared OPM- and SQUID-MEG systems in a subject group, with most limited to a small number of OPM sensors that do not provide whole-brain coverage^10,12,27,28^. Nevertheless, findings demonstrate similar source localization accuracy and higher amplitude and signal-to-noise ratio (SNR) responses in OPM-MEG relative to SQUID-MEG—with examples including visual gamma, visuo-motor, auditory, somatosensory tasks, and epileptic activity^10,12,27,28^; no study has investigated the replicability of emotional face processing using OPM-MEG. Moreover, this research has only investigated a single effect, while to our knowledge none have examined neural function and connectivity in the same study.

The present study investigated the evoked responses, oscillatory power modulation, and whole-brain functional connectivity to emotional faces in OPM- and SQUID-MEG in 15 adults, to ascertain the replicability across the two systems. Here, we extended previous work to include the largest number of participants to date, using 40 dual-axis OPM sensors (forming an 80-channel MEG system (Cerca Magnetics Limited, Nottingham, UK)), to record data during a visual protocol that is widely used in both basic and clinical research. Furthermore, we obtained data from five other participants who were incompatible with SQUID-MEG due to dental fixtures (SQUID-MEG-incompatible). We hypothesized comparable latency of evoked emotional face responses, spatial patterns of oscillatory power, and patterns of whole-brain connectivity between OPM- and SQUID-MEG modalities. Lastly, we expected to attain high-quality OPM-MEG recordings in SQUID-MEG-incompatible participants.

## Results

### Participants

OPM- and SQUID-MEG data were obtained from 16 SQUID-MEG-compatible adults (20–56 years of age; 9 males) and 5 SQUID-MEG-incompatible adults (22–69 years of age; 2 males) while they performed a passive emotional face processing task where they were presented with happy and angry faces. One SQUID-MEG-compatible male participant was excluded for being an outlier (> 3SD from the mean) with respect to the M170 response. Participant demographics are summarized in Table 1; there were no significant differences in age nor sex between the SQUID-MEG-compatible (n = 15) and—incompatible (n = 5) participants.

**Table 1:** Participant demographics.

Measure	Mean [SD]		Statistics^a^		
	SQUID-MEG-compatible	SQUID-MEG-incompatible	Test statistic	*p*-value	Effect size^b^
*N*	15	5	–	–	–
Age (years)	31.0 [9.0]	36.4 [18.9]	0.89	.387	.46
Sex					
Male	8	2	0.27	.606	1.71
Female	7	3			

*SD* standard deviation; ^a^*t*-test for continuous variables, and *Χ*^2^-test for categorical variables; ^b^Cohen’s d for continuous variables, and odds ratio for categorical variables.

After removing trials with artefacts, the total number of remaining trials and mean head motion across these trials was compared between the OPM- and SQUID-MEG modalities (Table 2). For the SQUID-MEG-compatible participants, there was no difference between the OPM- and SQUID-MEG modalities in the number of trials retained nor head motion. For the SQUID-MEG-incompatible participants, there was a significant increase in the number of trials retained for the OPM-MEG compared to the SQUID-MEG modality (*F* = 122.45, *p* < 0.001, *η*_*p*_^2^ = 0.97), with no trials retained after quality control for any of the incompatible participants in the SQUID-MEG system. Since no trials were retained, head motion could not be compared for the SQUID-MEG incompatible participants.

**Table 2:** Repeated measures ANOVA comparing the within-participant effect of modality (OPM, SQUID-MEG) for the data quality measures, separately for the SQUID-MEG-compatible and -incompatible participants.

	Measure	Mean [SD]		Statistics^a^		
		OPM-MEG	SQUID-MEG	*F*	*p*-value	*η*_*p*_^2^
SQUID-MEG-compatible	# trials	77.5 [6.0]	77.9 [2.7]	0.09	.766	.01
	Head motion (mm)	1.0 [0.3]	1.4 [1.3]	2.26	.157	.15
SQUID-MEG-incompatible	# trials	67.8 [13.7]	0.0 [0.0]	122.45	< .001	.97
	Head motion (mm)	0.9 [0.2]	–	–	–	–

*SD* standard deviation; OPM-MEG: optically- pumped magnetometer-magnetoencephalography; SQUID-MEG: superconducting quantum interference device-magnetoencephalography ^a^Repeated measures ANOVA.

### M170 face response in the OPM-MEG and SQUID-MEG systems

**Figure 1:**
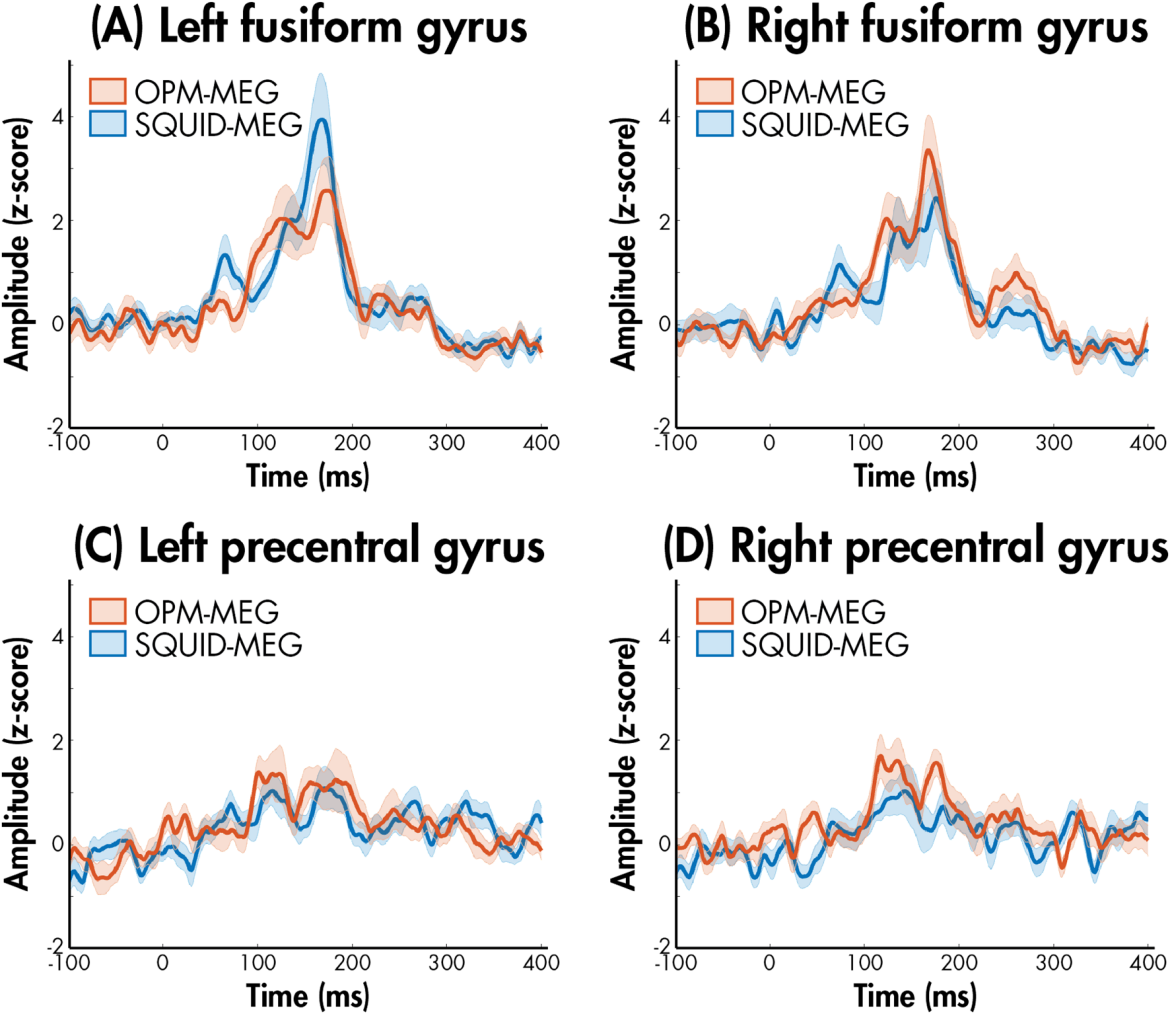
Source reconstructed timeseries for the OPM (red) and SQUID (blue) data for the left and right fusiform gyri (**A** and **B**, respectively) and the left and right precentral gyri (**C** and **D**, respectively), averaged across all SQUID-MEG-compatible participants; shaded standard error is also presented.

Neurophysiological timeseries were extracted for the bilateral fusiform gyri to examine the M170 evoked response to faces for the SQUID-MEG-compatible participants (Fig. 1A,B); timeseries for the precentral gyri are also presented as a control region (Fig. 1C,D). We observed the M170 peak response to emotional faces bilaterally in the fusiform gyri in both the OPM- and SQUID-MEG systems.

The peak amplitude and latency of the M170 response occurring between 120 and 180 ms was extracted; repeated measure ANOVAs were used to test for effects of hemisphere and modality (Table 3). There was no significant effect of hemisphere nor latency for either peak amplitude or latency.

**Table 3:** Repeated measures ANOVA examining the within-participant effects of hemisphere (right, left) and modality (OPM-MEG, SQUID-MEG) for the latency and amplitude of the M170 response in the fusiform for the SQUID-MEG-compatible participants.

Measure	Mean [SD]		Statistics^a^			
	OPM-MEG	SQUID-MEG	Term	*F*	*p*-value	*η*_*p*_^2^
*M170*						
Latency (ms)						
Right	157.3 [15.5]	152.6 [20.1]	Hemisphere	0.03	.870	< .01
Left	152.0 [20.2]	159.4 [12.6]	Modality	0.12	.738	< .01
Amplitude (nAm)						
Right	4.8 [2.6]	4.2 [2.0]	Hemisphere	0.65	.435	.01
Left	4.7 [2.2]	5.1 [2.8]	Modality	0.03	.861	< .01

*SD* standard deviation, *OPM-MEG* optically- pumped magnetometer-magnetoencephalography; *SQUID-MEG* superconducting quantum interference device-magnetoencephalography; ^a^Repeated measures ANOVA.

**Figure 2:**
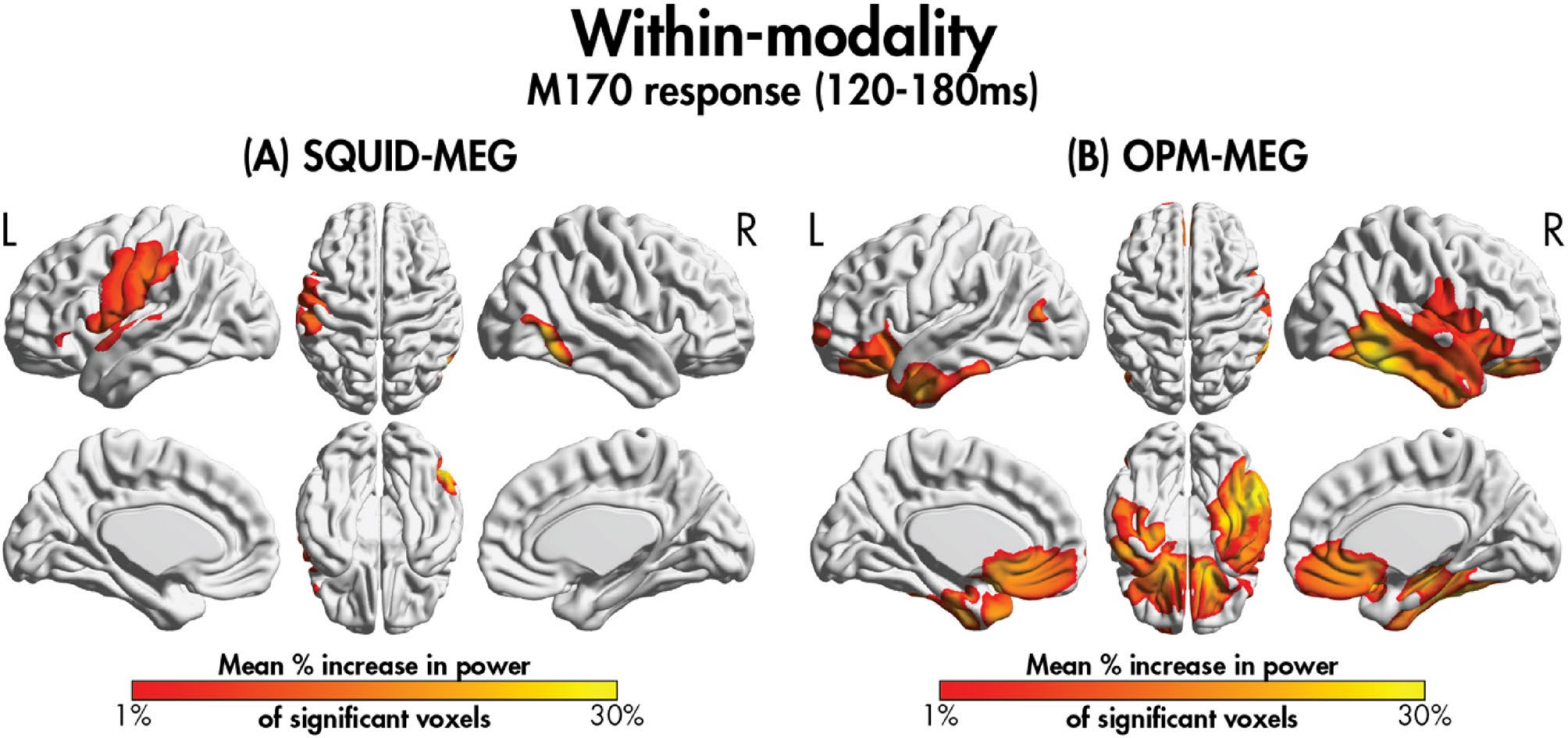
The percentage increase in power between 120 and 180 ms compared to baseline for significant voxels within the (**A**) SQUID data and (**B**) OPM-MEG data, averaged across SQUID-MEG-compatible participants.

To further characterize the M170 response, we computed the percentage increase in power for the active window (120–180 ms) compared to baseline (− 60 to 0 ms). Assessment of significant (*p*_FWE_ < 0.05) within-modality increases in power between 120–180 ms relative to baseline in both the SQUID and OPM systems showed spatially similar increases in power for the SQUID-MEG-compatible participants (Fig. 2). We calculated the percentage of significant voxel overlap with AAL regions, which showed increased power in the right fusiform gyri, the right middle and inferior temporal areas, the left pole of the superior temporal gyrus, the right inferior occipital gyri, the left insula, and left inferior frontal regions in both systems. For the OPM system, we found significantly increased power compared to baseline in several additional areas not seen in the SQUID-MEG system including the left fusiform gyrus, more extensive regions in the temporal lobes, the right insula, the bilateral amygdalae, the bilateral anterior cingulate cortices, and several bilateral frontal areas (see Supplemental Table 1 for a list of all regions). The percentage change in power 120–180 ms following the onset of emotional faces showed no significant differences between the OPM- and SQUID-MEG systems (*p*_FWE_ < 0.05).

### Functional connectivity to emotional faces in the OPM-MEG and SQUID-MEG modalities

**Figure 3:**
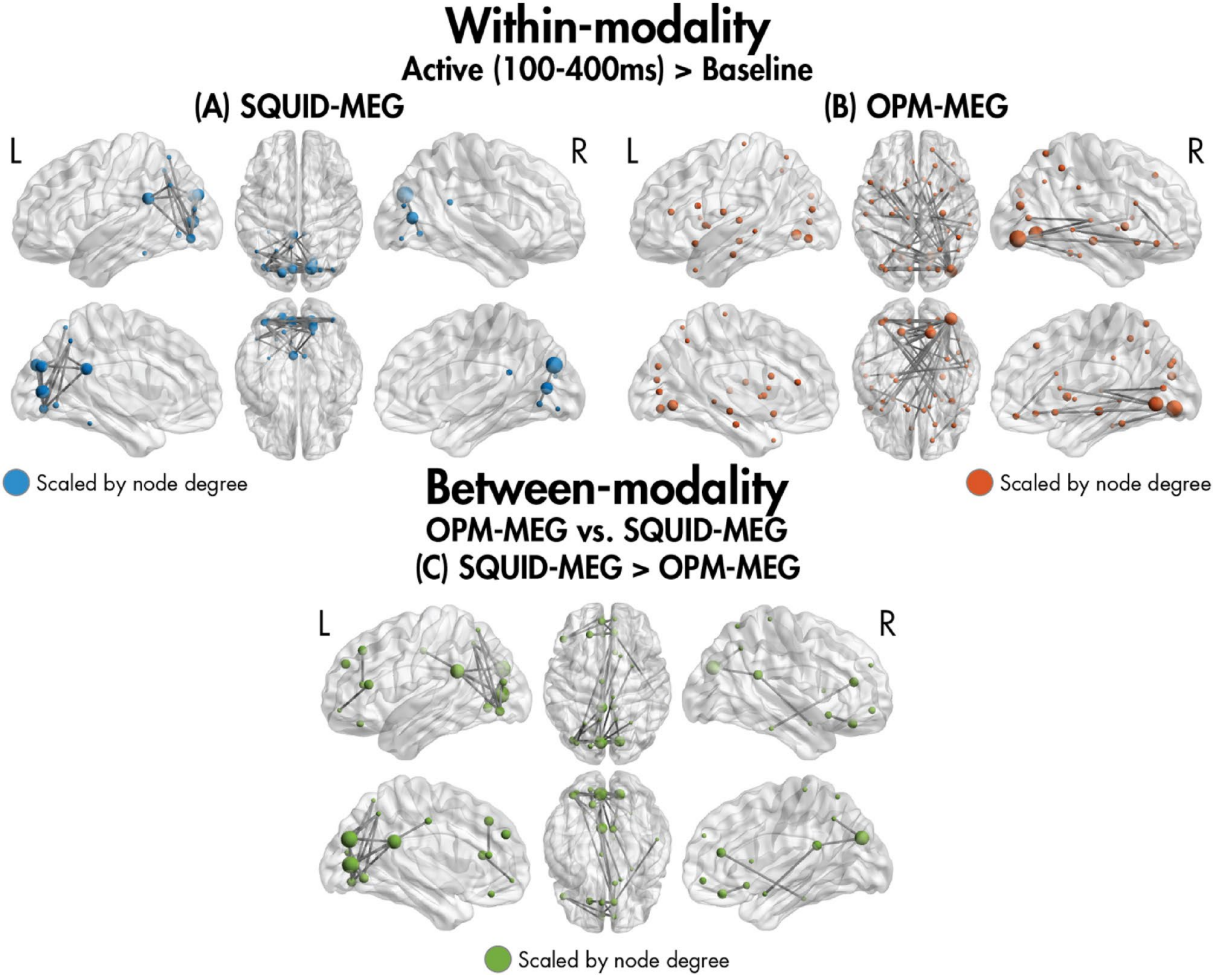
The networks showing significantly increased connectivity in the active window (100–400 ms) compared to baseline (− 300 to 0 ms) within the (**A**) SQUID and (**B**) OPM data in the SQUID-MEG-compatible participants, and the networks showing significant between-modality differences in this time window (**C**).

Phase-based functional connectivity was computed for an active window (100–400 ms) relative to baseline (− 300 to 0 ms). Significant (*p*_FWE_ < 0.05) within-modality increased connectivity in the active compared to baseline window was observed for both the SQUID and OPM systems for the SQUID-MEG-compatible participants (Fig. 3A,B). For the SQUID-MEG system (41 edges, 14 nodes, *p*_FWE_ < 0.001), we observed hubs in the occipital areas, including the bilateral middle, inferior and superior occipital gyri, lingual gyri, and cuneus, and the left fusiform gyrus, with many connections to other visual areas. For the OPM-MEG system (43 edges, 24 nodes, *p*_FWE_ < 0.001), we similarly saw hubs in the occipital areas, including the bilateral inferior and middle occipital gyri, and left lingual gyrus and cuneus. The network primarily involved connections between these regions and other occipital, limbic, parietal and temporal areas, such as the right superior temporal gyrus. When comparing between modalities (Fig. 3C), a network with significantly increased connectivity in the SQUID-MEG compared to OPM-MEG system was observed (41 edges, 21 nodes, *p*_FWE_ < 0.001). The network was anchored in the occipital lobe, with several connections between occipital and parietal areas.

### Comparable OPM-MEG data from SQUID-MEG-incompatible participants

**Figure 4:**
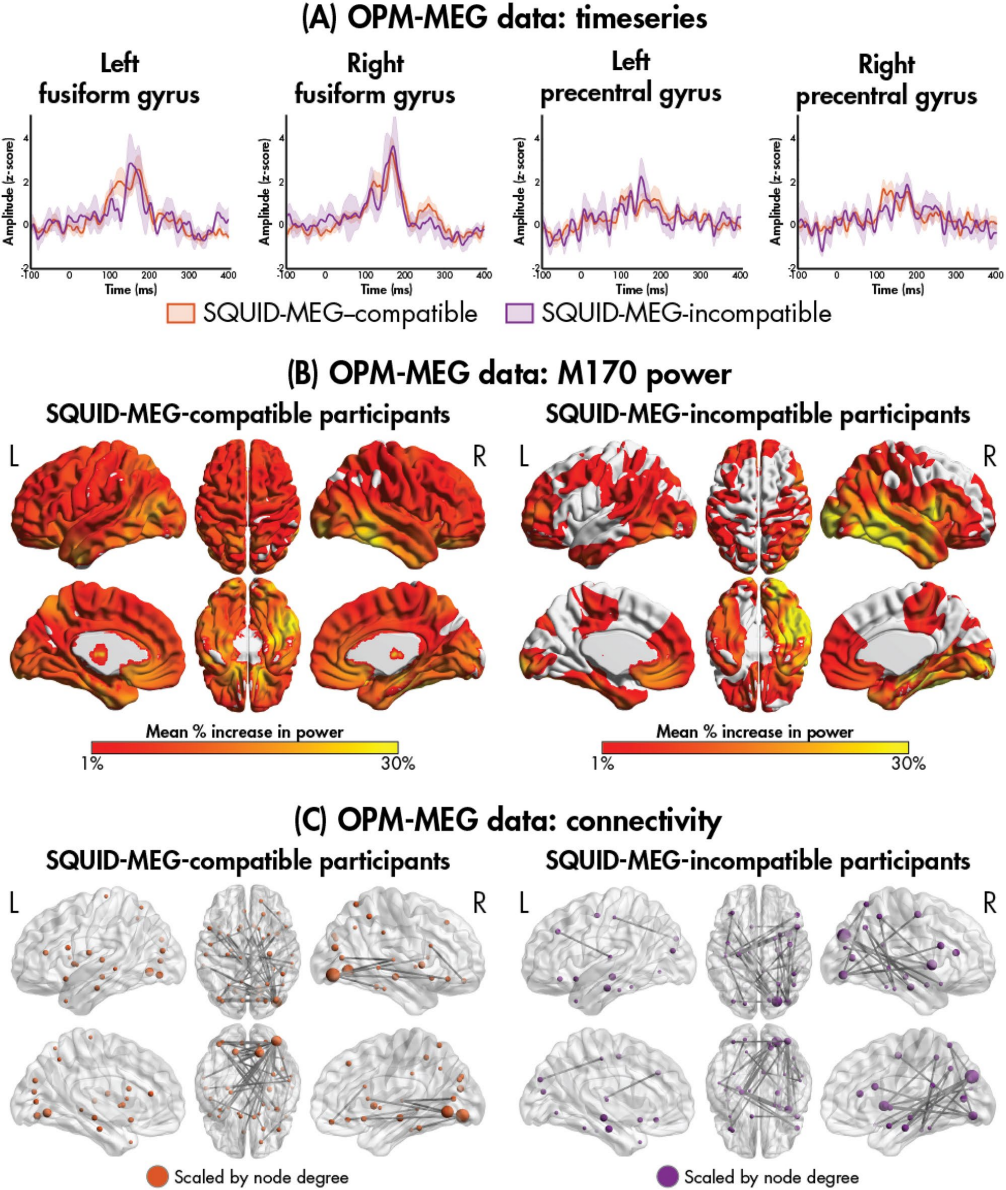
Comparison of the OPM-MEG M170 evoked timeseries (**A**), M170 power (**B**), and connectivity (**C**) data between the SQUID-MEG-compatible and -incompatible participants.

While no trials were retained for the SQUID-MEG data from the SQUID-MEG-incompatible participants, we visually compared the M170 and connectivity patterns between the SQUID-MEG-compatible and -incompatible participants for the OPM-MEG data (Fig. 4; Table 4). Due to the small sample size of the incompatible participants (*N* = 5), no within-modality statistics were performed for evoked responses and oscillatory power, and no between-modality statistics were performed. A comparable evoked M170 response was observed in the incompatible participants (Fig. 4A), with increases in oscillatory power and functional connectivity also showing similar spatial patterns and hub regions, respectively (Fig. 4B,C, respectively).

**Table 4:** Mean peak amplitudes and latencies for the M170 response in the fusiform gyri from the OPM data for the SQUID-MEG-compatible and -incompatible participants.

Measure	Mean [SD]	
	SQUID-MEG-compatible	SQUID-MEG-incompatible
*M170*		
Latency (ms)		
Right	157.3 [15.5]	157.5 [22.7]
Left	152.0 [20.2]	162.8 [14.1]
Amplitude (nAm)		
Right	4.8 [2.6]	5.7 [3.3]
Left	4.7 [2.2]	4.6 [3.1]

*SD* standard deviation, *OPM-MEG* optically pumped magnetometer-magnetoencephalography; *SQUID-MEG*, superconducting quantum interference device-magnetoencephalography.

## Discussion

The present study is the first to characterize and establish replicability of neurophysiological function and connectivity to emotional faces in OPM- and SQUID-MEG systems. We further extended previous work comparing the two systems by using a larger sample of participants and a whole-head 40-dual-axis channel OPM system. The well-established M170 face-sensitive response was localized to the fusiform gyri in both modalities. Similar patterns of activation to faces across the brain were also seen between the two modalities. Whole-brain functional connectivity contrasts revealed increased phase synchrony in an occipital-parietal anchored network in the SQUID compared to the OPM system, suggesting disproportionate coverage/sensitivity in the SQUID system. Notably, OPMs allowed for useable data recordings in participants with metallic dental work.

We observed a clear M170 face response bilaterally in the fusiform gyri in both the OPM- and SQUID-MEG systems. The peak ampltiudes and latencies across modalities were comparable, with no significant differences in modality. Thus, using OPM-MEG, we replicated the neurophysiological face-sensitive component. This is consistent with previous reports of comparable source-level brain signals during visuo-motor, visual, working memory and somatosensory tasks between SQUID and OPM-MEG systems^8–10^. For instance, Boto and colleagues^10^ found no apparent brain current difference between modalities for somatosensory stimulation.

As expected, there were no significant between-modality effects in the spatial patterns of regional power. When examining within-modality effects of power compared to baseline, we found an increase in activity in several cortical and subcortical areas, consistent with those activated during face processing tasks^29,30^, across the two different systems. For both systems, these regions primarily included occipital-temporal, limbic and frontal areas, including bilateral superior temporal gyri, fusiform gyri, insulae and inferior frontal gyri. However, the extent of spatial activation by the OPM-MEG was more widespread compared to the SQUID-MEG system, involving the recruitment of several additional face processing areas, suggesting higher signal-to-noise which aligns with a growing body of research^10,12,27^. Importantly, we also found greater activation in subcortical areas with the OPM system, further reinforcing the improved signal-to-noise to include deep brain sources. It is important to note the possibility that the more widespread spatial activation by the OPM- vs. SQUID-MEG could be also contributed by signal leakage among regions given the lower channel count. However, because the additional regions are known to support face processing, it is unlikely that signal leakage is the only driver of this finding.

A network of greater functional connectivity in SQUID-MEG compared to OPM-MEG was found involving connections anchored in visual regions that extended to other bilateral occipital, parietal, and limbic areas. It is possible that this SQUID-MEG-derived network of increased occipital and parietal connectivity was a result of the difference in sensor coverage between systems. Previous research has shown inhomogeneous signal sensitivity over the cortex with SQUID-MEG due to the uneven spacing between the head and the sensors, particularly when participants lay in the supine position as in the current study, resulting in higher signal strengths for occipital and parietal areas^11,15,16^. Thus, the SQUID-MEG system likely showed disproportionally enhanced sensitivity to connections in occipital and parietal areas compared to OPM-MEG, due to the participants being supine in the SQUID-MEG.

Remarkably, we demonstrated for the first time, the ability to obtain high-quality MEG recordings in all five participants with irremovable metal (i.e., dental work) using OPM-MEG, while no usable data were obtained using the SQUID-MEG system. Metallic implants produce significant magnetic interference in the SQUID-MEG caused by even tiny head movement in relation to the fixed sensor array^19^. In the OPM-MEG, because these metallic devices are fixed relative to the head-mounted sensors they do not produce significant noise^6,16^. In accordance with this, our findings revealed that the SQUID-MEG-incompatible participants had a significantly higher data retention (i.e., total number of trials) in the OPM than in the SQUID system. We also show that neural mechanisms to faces can be robustly characterized using OPM-MEG in participants with dental work. Visually, the data showed a clear evoked M170 peak to faces in the bilateral fusiform gyri. The SQUID-MEG-incompatible participants also showed similar patterns of regional power to emotional faces relative to baseline compared to the SQUID-MEG-compatible participants.

Despite the many strengths of this study, there are some limitations to consider. First, the data were collected in the supine position in the SQUID-MEG system while participants were sitting during the OPM-MEG data collection, which could have resulted in some of the differences in the distribution of the functional networks observed. Second, the screen was positioned closer to the participant in the SQUID-MEG compared to the OPM-MEG system, although the visual angle of the stimuli was equivalent between modalities. Third, we were not able to perform valid statistical contrasts comparing the M170 evoked response, regional power and functional connectivity between SQUID-MEG-incompatible and -compatible participants due to a small number of incompatible individuals. Thus, future work should replicate these analyses with larger and equivalently sized samples to determine statistical differences. Finally, we note that consistent with other OPM- and SQUID-MEG comparison studies, the current study does not provide an equivalent comparison between systems, given the differences in sensor counts between modalities. Since OPM-MEG is closer in proximity to the head than SQUID-MEG, the measured magnetic field patterns are more focal^6^, thus an OPM-MEG would benefit from a larger number of sensors. The development of OPM-MEG systems with greater channel counts will be an important avenue for future work.

In conclusion, this study demonstrates the strength and validity of measuring neurophysiological task-related responses using OPM-MEG in the largest number of participants to-date. We further establish the advantage of OPM-MEG in acquiring high quality recordings with participants with metallic implants, such as dental work, from whom no useable data were collected in the conventional system. These foundational findings can be leveraged for future studies of brain function and connectivity, particularly in very young children or clinical groups that could otherwise not be scanned using conventional MEG systems due to difficulties with staying still (e.g., individuals with epilepsy or movement disorders).

## Methods

### Participants

OPM- and SQUID-MEG data were obtained from 16 SQUID-MEG-compatible adults (20–56 years of age; 9 males) and 5 SQUID-MEG-incompatible adults (22–69 years of age; 2 males) at the Hospital for Sick Children in Toronto, Canada. One SQUID-MEG-compatible male participant was excluded for being an outlier (> 3SD from the mean) with respect to the M170 response, thus data were included in the analyses from 15 SQUID-MEG-compatible adults. Written informed consent was obtained from all participants, and the study was approved by the Hospital for Sick Children research ethics board and was performed in accordance with relevant guidelines and regulations.

### Experimental paradigm

An emotional faces task was completed by each participant using both the OPM- and SQUID-MEG systems, counterbalancing the order of the two systems. Each trial consisted of an emotional face (happy or angry faces from the NimStim Set of Facial Expressions^31^ presented for 500 ms; each trial was followed by an inter-stimulus interval (i.e., fixation cross) with a jittered duration (1250 ± 200 ms). A total of 80 randomized trials (40 happy, 40 angry) were presented using Presentation software (Neurobehavioural Systems, California, USA).

### Data acquisition

The OPM-MEG system consisted of an array of 40 dual-axis zero-field magnetometers (providing 80 channels) (QuSpin Inc., Colorado, USA) integrated into a whole-head wearable MEG system (Cerca Magnetics Limited). The sensors were mounted in one of two 3D-printed rigid helmets, depending on the participant’s head size, and were evenly distributed across the helmet to provide whole-head coverage. The participant was seated wearing the helmet in a magnetically shielded room (MSR; Vacuumschmelze, Hanau, Germany), at the centre of a set of bi-planar nulling coil panels (Cerca Magnetics Limited) and between a reference array of OPM sensors, to keep the sensors operating within ± 3.5nT by dynamically compensating the background magnetic field and its drift over time (for further details, see Hill et al., 2022; Holmes et al., 2018, 2019; Rea et al., 2021). A four camera system (OptiTrack Flex 13, NaturalPoint Incorporated, Oregon, USA) with infra-red markers placed on the bi-planar coils and helmet was used to continuously track head movement. Data from each channel were recorded using a digital acquisition system (National Instruments, Texas, USA) with a 1,200 Hz sampling rate. Prior to obtaining the task data, an empty room noise recording was acquired with no participant present. A three-dimensional optical imaging system consisting of a laptop, structure sensor camera (Occipital Incorporated, California, USA), and Skanect software (Occipital Incorporated) was used to obtain the position of the OPM sensors relative to the head.

For the SQUID-MEG data, a 151-channel first order axial gradiometer CTF MEG system (CTF MEG International Services LP, Coquitlam, Canada) along with synthetic third-order gradiometer noise cancelation was used. The system was housed in a VAC MSR (Vacuumschmelze, Hanau, Germany), and data were collected while participants were lying in the supine position. Fiducial coils placed on the participants’ nasion and bilateral preauricular points were used to continuously track head movement. Data were recorded at a 600 Hz sampling rate with an online antialiasing low-pass filter (150 Hz) and third-order spatial gradient noise cancellation.

### Preprocessing

All preprocessing was performed using MATLAB (MathWorks, Massachusetts, USA) using the FieldTrip toolbox (v 20,220,214)^32^ and custom scripts. For both the OPM- and SQUID-MEG data, the preprocessing pipeline consisted of the identification of noisy channels, filtering, trial epoching, independent component analysis (ICA), and trial rejection.

To be consistent across the two modalities, noisy channels were quantitatively identified in an iterative procedure using the power spectral density (PSD). First, for each channel, the median PSD across 10-s epochs was calculated, using the raw data from 1 to 150 Hz with 1 Hz intervals. To exclude frequency intervals corresponding to noise (e.g., power line noise), the median PSD across all channels was computed and frequency intervals that were more than three scaled median absolute deviations from the median were removed (MATLAB’s *isoutlier* function). Next, noisy channels were identified as being outliers in more than 75% of the remaining frequency intervals and removed from the data. Finally, this procedure was iteratively performed until no additional noisy channels were identified; for the OPM-MEG data, homogeneous field correction (HFC;^33,34^ was first performed using the data from the remaining channels at the start of each iteration to remove external interference modelled as a spatially constant homogeneous magnetic field.

The OPM- and SQUID-MEG data from the remaining channels were bandpass filtered between 1 and 150 Hz (4th order, two-pass Butterworth) and band-stop filtered to remove environmental noise. For the SQUID-MEG data, the 60 Hz power line noise and its harmonics were band-stop filtered (4th order, two-pass Butterworth, 2 Hz width). For the OPM-MEG data, additional filter frequencies were required due to interference from electrical equipment, including the OptiTrack cameras (~ 8 to 9 Hz and its harmonics), as well as disturbances from the OPM-MEG power supply (10 Hz and its harmonics). To objectively identify these frequencies, the empty room noise data were used. After identifying bad channels from the noise data, epoching, and bandpass filtering, the median PSD across all epochs and channels was computed for both the task and noise data; only peaks identified (MATLAB’s *findpeaks* with a peak prominence of 3 fT^2^/Hz) in both datasets were selected. The data were then band-stop filtered (4th or 3rd order, two-pass Butterworth), with the width set to be the minimum width for which the peak was no longer identified. Independent component analysis (ICA) was applied, using visual inspection to remove ocular and cardiac artefacts, and the data were epoched into trials − 1 s to 1.5 s relative to stimulus onset.

Trial rejection was then performed to identify trials with MEG signal that contained artefacts. For SQUID-MEG, this is typically done by excluding trials with signals that exceed a magnetic field strength threshold (*T*_cryo_, typically of 2000 fT^35^. However, this threshold needs to be adjusted for the OPM MEG data given the higher noise floor^6^. To objectively determine the adjusted OPM threshold (*T*_opm_) to be consistent across the two MEG modalities, we computed a ratio of the variance in magnetic field strength for the OPM-MEG versus the SQUID-MEG data and used this ratio to inflate the SQUID-MEG threshold. Specifically, for each of the SQUID-MEG-compatible participants, we excluded trials from the SQUID-MEG data with signal > 2000 fT and computed the median standard deviation of magnetic field strength across sensors and trials (*SD*_cryo_). We then computed the median standard deviation across all trials for the OPM-MEG data (*SD*_opm_), computed the ratio (*r* = *SD*_opm_/*SD*_cryo_), and multiplied the SQUID-MEG threshold by this ratio to obtain the OPM-MEG threshold (*T*_opm_ = 2000 × *r*). We applied this threshold to the OPM-MEG data to exclude artefactual trials and repeated this procedure until the threshold stabilized at 27,568 fT. To measure head motion, the maximum displacement from the median head position was computed for each included trial.

### Source reconstruction

While individual T1-weighted MRIs are typically used to determine the position of the sensors relative to brain anatomy, a necessity for source reconstruction, acquiring such images can be costly, timely, and difficult to acquire on some populations such as young children and clinical cohorts; using head and source models derived from template MRIs has been shown to yield comparable results to participant-specific head models^36,37^. Thus, for both modalities, the ICBM 152 standard space T1-weighted magnetic resonance image (MRI)^38^ was used to construct a single-shell head model^39^ and 2-mm grid source model restricted to the gray matter. The head and source models were then spatially realigned to the MEG data in subject-space. For the OPM-MEG data, this was performed by first surface matching the digitisations of the participants head and face with and without wearing the helmet, followed by surface matching to the template’s scalp surface. For the SQUID-MEG data, this was performed by co-registering the fiducials placed at the participants’ nasion and bilateral preauricular points with the nasion and preauricular points on the template MRI.

The OPM-MEG and SQUID-MEG data were further bandpass filtered between 2 and 40 Hz (4th order, two-pass Butterworth). A covariance matrix was computed across data from the entire task (excluding rejected trials^40^, and regularized using the Tikhonov method^41^, setting the regularization parameter to 2% of the unregularized matrix’s maximum eigenvalue. Forward solutions for each grid voxel were computed using the single-shell head models with a dipole approximation of neural current. For both modalities, source reconstruction was performed across the 2-mm grid using a linearly constrained minimum variance (LCMV) beamformer^42^. Using the beamformer weights from each voxel, the percentage increase in oscillatory power between active and baseline windows can be computed. To reconstruct electrophysiological activity for each cortical and subcortical region of the Automated Anatomical Labelling (AAL) atlas^43^, the timeseries for the voxel within each parcel showing maximum increase in power between 100–400 ms, a time window known to capture emotional face processing^44–46^, compared to baseline (− 0 to 300 ms) was extracted. The source reconstructed data were projected to the same dominant orientation across all participants for both modalities, such that we computed a set of weights for each tangential component of the dipole, and then computed the square-root of the sum-of-squares to get one timeseries for each participant. We transformed the OPM-MEG and SQUID-MEG timeseries data into a standard metric by z-scoring, which allowed us to accurately compare the amplitudes across both different modalities.

### M170 evoked response and oscillatory power

The bilateral fusiform gyri are core areas involved in face and emotional face perception^47–50^ and show a robust face response at approximately 170 ms, which is known as the M170 component in MEG; typically, larger in the right hemisphere^51,52^. To characterize this evoked response, the peak amplitude and latency were extracted from the fusiform gyri timeseries for each hemisphere within a 120–180 ms time window. While the fusiform gyri are classically associated with the M170 response, a wider network of regions involved in face processing, such as the bilateral occipital areas, and superior temporal gyri are also recruited during early stages of face processing^52,53^. Thus, we also computed the percentage increase in broadband oscillatory power between 120–180 ms compared to baseline (− 60 to 0 ms) for each voxel across the brain.

### Functional connectivity

Measures of functional connectivity have been used to characterize emotional face processing, showing the engagement of a network of brain regions including the bilateral fusiform gyri, superior temporal gyri, anterior cingulate cortices, insulae, amygdalae and orbital frontal brain areas, within a 100–400 ms window relative to stimulus onset in adults^54,55^. Thus, we computed phase-based connectivity networks for each participant. The Hilbert transform was applied to obtain timeseries of instantaneous phase for each AAL region, and pairwise phase synchrony was computed using the cross-trial weighted phase lag index (wPLI)^56^; the pairwise timeseries of connectivity values were *z*-scored relative to a baseline window (− 300 to 0 ms) and averaged across the active window (100–400 ms).

### Statistics

Differences between the SQUID-MEG-compatible and SQUID-MEG-incompatible participants in age were evaluated using a *t*-test with Cohen’s *d* effect size^57^; differences in sex were evaluated using a chi-squared test with odds ratios for effect size. Repeated measures ANOVAs were used to compare the within-participant effect of modality (OPM-MEG and SQUID-MEG) for the number of included trials and mean head motion across the included trials, separately for the SQUID-MEG-compatible and -incompatible participants, reporting partial eta-squared effect sizes.

For the M170 evoked response latency and amplitude, repeated measures ANOVAs were used to examine the within-participant effects of hemisphere (right and left) and modality (OPM-MEG and SQUID-MEG), reporting partial eta-squared effect sizes. For the voxel-wise measures of oscillatory power, significant within-modality increases in the percentage change of power compared to baseline were tested using non-parametric one-sample *t*-tests (5,000 permutations; FMRIB Software Library’s *randomise*^58^), using threshold-free cluster enhancement to control for the family-wise error (FWE) rate and holding significance at *p*_FWE_ < 0.05; paired *t*-tests were used to probe for between-modality differences in the percentage change of power compared to baseline. For connectivity, significant within-modality increases during the active window (100–400 ms) were tested using non-parametric one-sample *t*-tests (5,000 permutations), using Network Based Statistics (NBS)^59,60^ to control for the FWER rate with a primary component-forming threshold such that 1% of possible connections remain, and holding significance at *p*_FWE_ < 0.05; paired *t*-tests were also used to examine between-modality differences. Statistics were only performed for the cryo-compatible participants; given the small sample size of the SQUID-MEG-incompatible participants, responses were only visualized.

### Supplementary Information

Supplementary Tables.

## Data Availability

The datasets generated during and/or analysed during the current study are available from the corresponding author on reasonable request.
