# Supplementary material for: Using optically pumped magnetometers to replicate task-related responses in next generation magnetoencephalography
Source: Sci Rep. 2024 Mar 18;14:6513. doi: 10.1038/s41598-024-56878-6 (PMC10948796; doi:10.1038/s41598-024-56878-6)
Supplement: Supplementary file 1 — Supplementary Tables. [file 41598_2024_56878_MOESM1_ESM.docx]

**Supplemental Tables**

**OPM-MEG data, SQUID-MEG-compatible participants**

**Supplemental Table 1:** Percentage overlap of significant voxels with AAL regions

| **Percentage** | **AAL Regions** |
| --- | --- |
| 10.85 | Temporal_Inf_R |
| 9.88 | Temporal_Mid_R |
| 5.96 | Fusiform_R |
| 5.90 | Temporal_Sup_R |
| 4.84 | Temporal_Inf_L |
| 4.19 | Insula_R |
| 4.15 | Frontal_Inf_Orb_L |
| 3.10 | Frontal_Inf_Orb_R |
| 2.98 | Rolandic_Oper_R |
| 2.70 | Rectus_L |
| 2.61 | Rectus_R |
| 2.44 | Frontal_Sup_Orb_L |
| 2.39 | Temporal_Pole_Sup_R |
| 2.26 | Hippocampus_R |
| 2.26 | ParaHippocampal_R |
| 2.13 | Frontal_Med_Orb_R |
| 2.06 | Frontal_Sup_Orb_R |
| 1.94 | Frontal_Med_Orb_L |
| 1.81 | Temporal_Pole_Mid_L |
| 1.78 | Temporal_Pole_Sup_L |
| 1.73 | Caudate_L |
| 1.65 | Temporal_Pole_Mid_R |
| 1.49 | Temporal_Mid_L |
| 1.47 | Fusiform_L |
| 1.45 | Cingulum_Ant_L |
| 1.42 | Putamen_L |
| 1.33 | Insula_L |
| 1.18 | ParaHippocampal_L |
| 1.14 | Postcentral_R |
| 1.14 | Cingulum_Ant_R |
| 1.13 | Caudate_R |
| 1.07 | Frontal_Inf_Oper_R |
| 1.01 | Olfactory_L |
| 0.92 | Olfactory_R |
| 0.90 | Putamen_R |
| 0.71 | Frontal_Mid_Orb_L |
| 0.59 | Heschl_R |
| 0.58 | Frontal_Mid_Orb_R |
| 0.49 | Pallidum_L |
| 0.35 | Frontal_Inf_Tri_R |
| 0.31 | Occipital_Inf_R |
| 0.27 | Frontal_Sup_L |
| 0.23 | Frontal_Sup_Medial_R |
| 0.23 | Frontal_Sup_Medial_L |
| 0.22 | Precentral_R |
| 0.20 | Hippocampus_L |
| 0.19 | Amygdala_L |
| 0.11 | Occipital_Mid_L |
| 0.09 | Amygdala_R |
| 0.06 | Frontal_Inf_Tri_L |
| 0.03 | Frontal_Mid_L |
| 0.02 | Lingual_R |
| 0.02 | Angular_R |
| 0.02 | Pallidum_R |
| 0.01 | Frontal_Sup_R |

.

**SQUID-MEG data, SQUID-MEG-compatible participants**

**Supplemental Table 2:** Percentage overlap of significant voxels with AAL regions

| **Percentage** | **AAL Regions** |
| --- | --- |
| 34.54 | Postcentral_L |
| 13.76 | Precentral_L |
| 11.71 | Rolandic_Oper_L |
| 8.98 | Temporal_Inf_R |
| 5.82 | Parietal_Inf_L |
| 5.34 | Temporal_Mid_R |
| 4.62 | Temporal_Sup_L |
| 4.24 | SupraMarginal_L |
| 4.14 | Frontal_Inf_Oper_L |
| 2.13 | Temporal_Pole_Sup_L |
| 1.80 | Insula_L |
| 1.28 | Frontal_Inf_Orb_L |
| 0.75 | Frontal_Inf_Tri_L |
| 0.66 | Heschl_L |
| 0.21 | Occipital_Inf_R |
| 0.02 | Fusiform_R |
